# Open-source statistical and data processing tools for wide-field optical imaging data in mice

**DOI:** 10.1117/1.NPh.10.1.016601

**Published:** 2023-03-01

**Authors:** Lindsey M. Brier, Joseph P. Culver

**Affiliations:** a Washington University School of Medicine, Department of Radiology, St. Louis, Missouri, United States; b Washington University School of Arts and Science, Department of Physics, St. Louis, Missouri, United States; c Washington University School of Engineering, Department of Biomedical Engineering, St. Louis, Missouri, United States; d Washington University School of Engineering, Department of Electrical and Systems Engineering, St. Louis, Missouri, United States

**Keywords:** calcium imaging, wide-field imaging, optical imaging, data processing

## Abstract

**Significance:**

Wide-field optical imaging (WOI) can produce concurrent hemodynamic and cell-specific calcium recordings across the entire cerebral cortex in animal models. There have been multiple studies using WOI to image mouse models with various environmental or genetic manipulations to understand various diseases. Despite the utility of pursuing mouse WOI alongside human functional magnetic resonance imaging (fMRI), and the multitude of analysis toolboxes in the fMRI literature, there is not an available open-source, user-friendly data processing and statistical analysis toolbox for WOI data.

**Aim:**

To assemble a MATLAB toolbox for processing WOI data, as described and adapted to combine techniques from multiple WOI groups and fMRI.

**Approach:**

We outline our MATLAB toolbox on GitHub with multiple data analysis packages and translate a commonly used statistical approach from the fMRI literature to the WOI data. To illustrate the utility of our MATLAB toolbox, we demonstrate the ability of the processing and analysis framework to detect a well-established deficit in a mouse model of stroke and plot activation areas during an electrical paw stimulus experiment.

**Results:**

Our processing toolbox and statistical methods isolate a somatosensory-based deficit 3 days following photothrombotic stroke and cleanly localize sensory stimulus activations.

**Conclusions:**

The toolbox presented here details an open-source, user-friendly compilation of WOI processing tools with statistical methods to apply to any biological question investigated with WOI techniques.

## 1 Introduction

Functional neuroimaging has enhanced our study of systems neuroscience and understanding of neural networks.^1^^,^^2^ Mainly, this has been accomplished with blood oxygen level-dependent (BOLD) fluctuations in functional magnetic resonance (fMRI) data in human subjects.^3^ In order to better understand human conditions, there has been an increase in functional neuroimaging in animal models, also performed using fMRI.^4^^–^^6^ However, the relatively small size of the mouse brain offers multiple technical and logistical challenges with fMRI. Therefore, there has been a parallel development of wide-field optical imaging (WOI) techniques in the mouse, yielding similar blood-based surrogates of neural activity at a similar spatial scale with various logistical tradeoffs versus fMRI.^7^ The advent of genetically encoded calcium indicators (GECIs) enables cell-specific labeling and led to increased temporal resolution for WOI compared to traditionally measured hemodynamics.^8^^–^^10^ Combined hemoglobin and fluorophore imaging is readily available with optical imaging systems and harnesses the advantages of GECIs as well as maintains a translatable blood-based recording directly comparable to human fMRI. WOI analysis faces many of the same procedural steps and therefore difficulties as those experienced with fMRI analysis, such as data processing, visualization, and statistical testing. However, the relative novelty of WOI compared to fMRI means that there is a need for many of the solutions within the fMRI community to be translated into the WOI data analysis communities. We have developed a toolbox (MATLAB) that addresses a number of fundamental concerns.

One of the biggest statistical challenges within the functional neuroimaging community is the problem of correcting for multiple statistical tests. Many solutions have been proposed within the fMRI community,^11^^,^^12^ however, in general these have not been translated into an easy-to-use WOI toolbox. Historically, functional connectivity (FC) is examined using a seed-based approach.^13^ For seed-based maps, common practice includes performing a pixel or voxel-wise statistical test (e.g., Student’s t) resulting in thousands of tests being performed within the field-of-view (FOV). The most stringent correction (i.e., Bonferroni) assumes each statistical test is independent.^14^ This is certainly not the case when examining neighboring pixels within a brain region for multiple reasons. For most mesoscopic WOI instruments, blurring by tissue light scattering brings the effective full-width half-maximum (FWHM) to a size that spans multiple pixels thus rendering each pixel not independent from an instrumentation point of view. Additionally, from a biological point of view, it is reasonable to assume an amount of dependence between neighboring pixels within the same brain region. A more plausible approach to handle the multiple comparisons problem that has become fairly standard for fMRI is the use of a clustering analysis, coupled with random field theory, to weight larger regions of interest (ROIs; i.e., large clusters) of contiguous neighboring significant pixels as more likely to be a statistically significant finding than small ROIs.^15^^,^^16^ In this paper, we translate the clustering approach to WOI pixel space application.

Here, we provide a mouse optical data processing toolbox to streamline and make processing steps transparent and user-friendly. Within it, we adopt the fMRI cluster size-based approach to determining statistical significance and apply it to wide-field optical FC mapping. We demonstrate the utility of this toolbox and various analytical packages within the context of photothrombotic stroke and sensory stimulus activations.

## 2 Methods

### 2.1 Animals

Four 3- to 4-month-old mice (two male, two female) were imaged at baseline (Day 0) and on Day 3 post photothrombotic stroke to left somatosensory forepaw cortex. All mice were *Thy1*-GCaMP6f [Jackson Laboratories Strain: C57BL/6J-*Tg(Thy1-GCaMP6f)GP5*.*5Dkim*; stock: 024276]. These mice express the protein GCaMP6f in excitatory neurons, primarily in cortical layers ii, iii, v, and vi.^8^ All studies were approved by the Washington University School of Medicine Animals Studies Committee and follow the guidelines of the National Institutes of Health’s Guide for the Care and Use of Laboratory Animals.

### 2.2 Surgical Preparations

Prior to imaging, typical surgical preparations were implemented.^9^^,^^17^ Briefly, isoflurane anesthesia (3% induction, 1% maintenance, 0.5  L/min) was used for sedation and an optically transparent 14×18  mm plexiglass window was implanted with translucent dental cement (C&B-Metabond, Parkell Inc., Edgewood, New York) following a midline incision and clearing of skin and periosteal membranes. The window covered the majority of the dorsal cortical surface and provided an anchor for head fixation and allowed for chronic, repeatable imaging.

### 2.3 Photothrombosis

Mice were secured in a stereotaxic frame under isoflurane anesthesia. 200  μL of Rose Bengal (Sigma Aldrich) dissolved in saline (10  g/L) was injected intraperitoneally. After 4 min, a 532-nm diode-pumped solid-state laser (Shanghai Laser & Optics Century) was focused to 2.2 mm left and 0.5 mm anterior to bregma with a 0.5 mm spot size and at 23 mW for 10 min.^18^ Mice were imaged at baseline [i.e., prior to photothrombosis (Day 0)], and 72 h post (Day 3). The dataset used in the following analyses consists of two five-minute imaging runs from each mouse. The stroke data were processed and analyzed as described below. Calcium data were filtered with a 0.4 to 4.0 Hz Butterworth bandpass filter and hemoglobin data with a 0.009 to 0.08 Hz Butterworth bandpass filter. These frequency bands were selected as they correspond to delta (0.4 to 4.0 Hz) and infraslow (0.009 to 0.08 Hz) ranges. The canonical FC frequency band (infraslow, 0.009 to 0.08 Hz) was used for hemoglobin-based analysis similar to the blood oxygen level dependent (BOLD) analysis used in the fMRI community.

### 2.4 Fluorescence and Optical Intrinsic Signal (OIS) Imaging

Mice were head-fixed in a stereotaxic frame and body secured in a black felt pouch for imaging. Sequentially firing LEDs (Mightex Systems, Pleasanton, California) passed through a series of dichroic lenses (Semrock, Rochester, New York) into a liquid light guide (Mightex Systems, Pleasanton, California) that terminated in a 75 mm f/1.8 lens (Navitar, Rochester, New York) to focus the light onto the dorsal cortical surface. LEDs consisted of 470 nm (GCaMP6f excitation), 530, 590, and 625 nm light. An sCMOS camera (Zyla 5.5, Andor Technologies, Belfast, Northern Ireland, United Kingdom) coupled with an 85 mm f/1.4 camera lens (Rokinon, New York, New York) was used to capture fluorescence/reflectance produced at 16.8 Hz per wavelength of LED. A 515 nm longpass filter (Semrock, Rochester, New York) was used to discard GCaMP6f excitation light. Cross polarization (Adorama, New York, New York) between the illumination lens and collection lens discarded artifacts due to specular reflection. The field-of-view (FOV) recorded covered the majority of the convexity of the cerebral cortex ($∼1.1\;{\mathrm{cm}}^{2}$), extending from the olfactory bulb to the superior colliculus. All imaging data were binned in 156×156  pixel^2^ images at $∼100\;{\mu m}^{2}$ per pixel.

### 2.5 Toolbox Capabilities and Workflow: Data and User Input

**Fig. 1:**
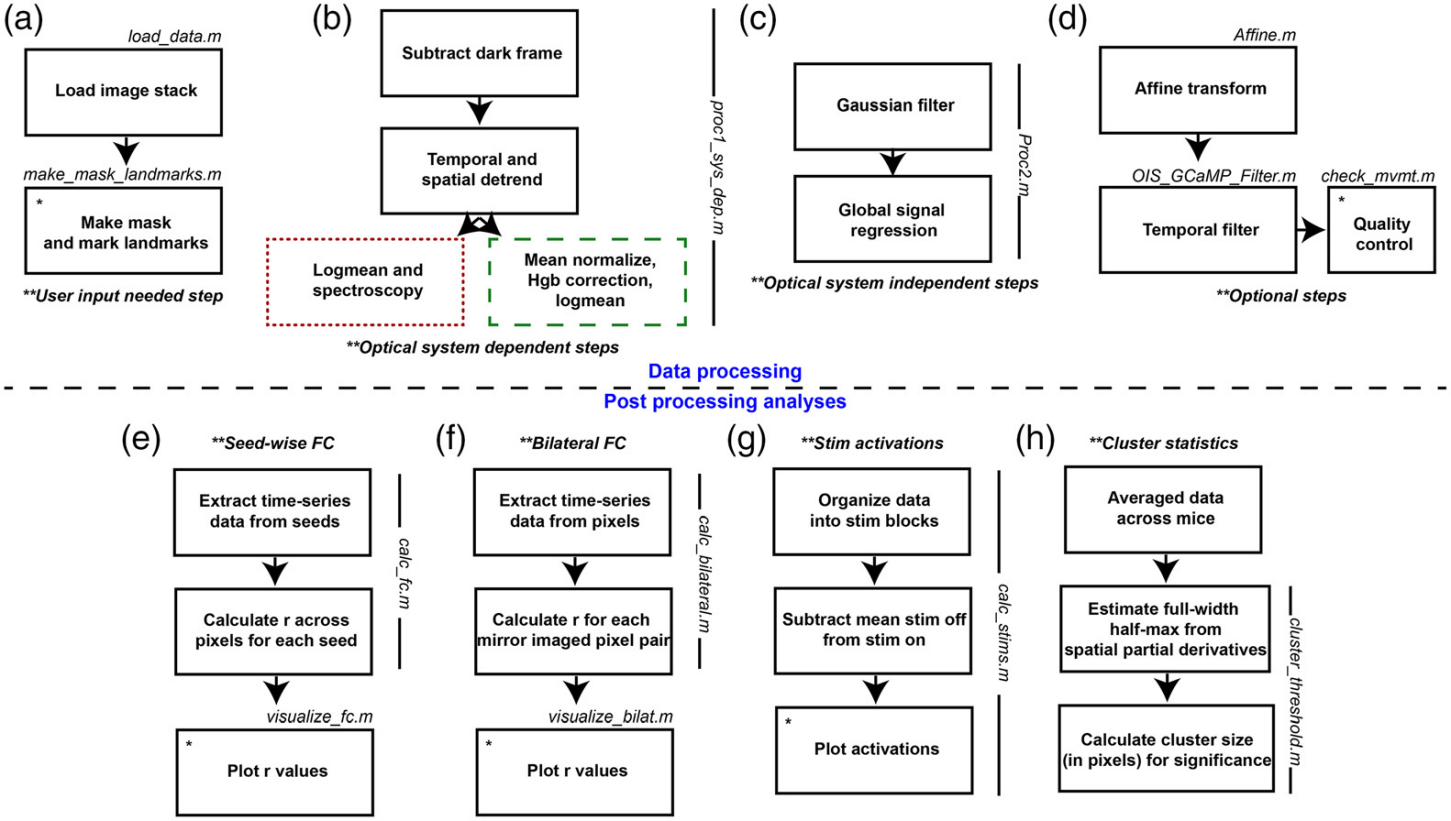
WOI mouse toolbox flowchart. (a) User input is needed after loading data, MATLAB will prompt user to trace the dorsal cortical surface as well as select anatomical landmarks. (b) Optical system dependent processing steps. These processing steps depend on the amount of ambient light present, natural fluctuations, and LEDs used for spectroscopy. (c) Optical system independent steps. (d) Optional steps such as affine transform if comparing multiple mice, temporal filtering, or quality control outputs. (e) Seed-wise FC analysis steps. (f) Bilateral FC analysis steps. (g) Stimulus activation analysis steps. (h) Cluster-based statistics steps used to find cluster size threshold in number of pixels.

In order to initiate use of the toolbox, data has to be loaded into MATLAB (we recommend version 2022a or newer). Data should be in the form of pixels by pixels by frames. Example data used in the following analyses were acquired using the aforementioned mesoscopic calcium imaging modality, however, usage can be expanded to incorporate any data configured into this data stack (e.g., voltage-sensitive dye imaging, data from other animal models). However, one caveat to be noted is the following data processing pipeline was optimized on mesoscopic WOI mouse data. All inputs and outputs to specific scripts mentioned and highlighted in Fig. 1 are specified in the header of each script. The explanation that follows will walkthrough each processing step, which are all in separate subroutines. Subroutines will be referenced, and scripts that should be edited to run by the user are highlighted in Fig. 1 and Table 1.

**Table 1:** Data processing functions to be run by user. Functions outlined at each processing step in Figs. 1(a)–1(d).

Function	Description
**load_data.m**	Load image stack
**make_mask_landmarks.m**	Uses roipoly.m, a built-in MATLAB function, to trace/create binary mask. User then clicks anterior suture and lambda to create seeds
**Proc1_sys_dep.m**	Data processing, image system dependent steps (uses Mightex and prahl .txt files for spectroscopy, examples on GitHub)
**Proc2.m**	Data processing, image system independent steps
**Affine.m**	Perform affine transform to common atlas space
**OIS_GCaMP_Filter.m**	Butterworth filtering
**check_mvmt.m**	Outputs the global variance of the temporal derivatives

**Fig. 2:**
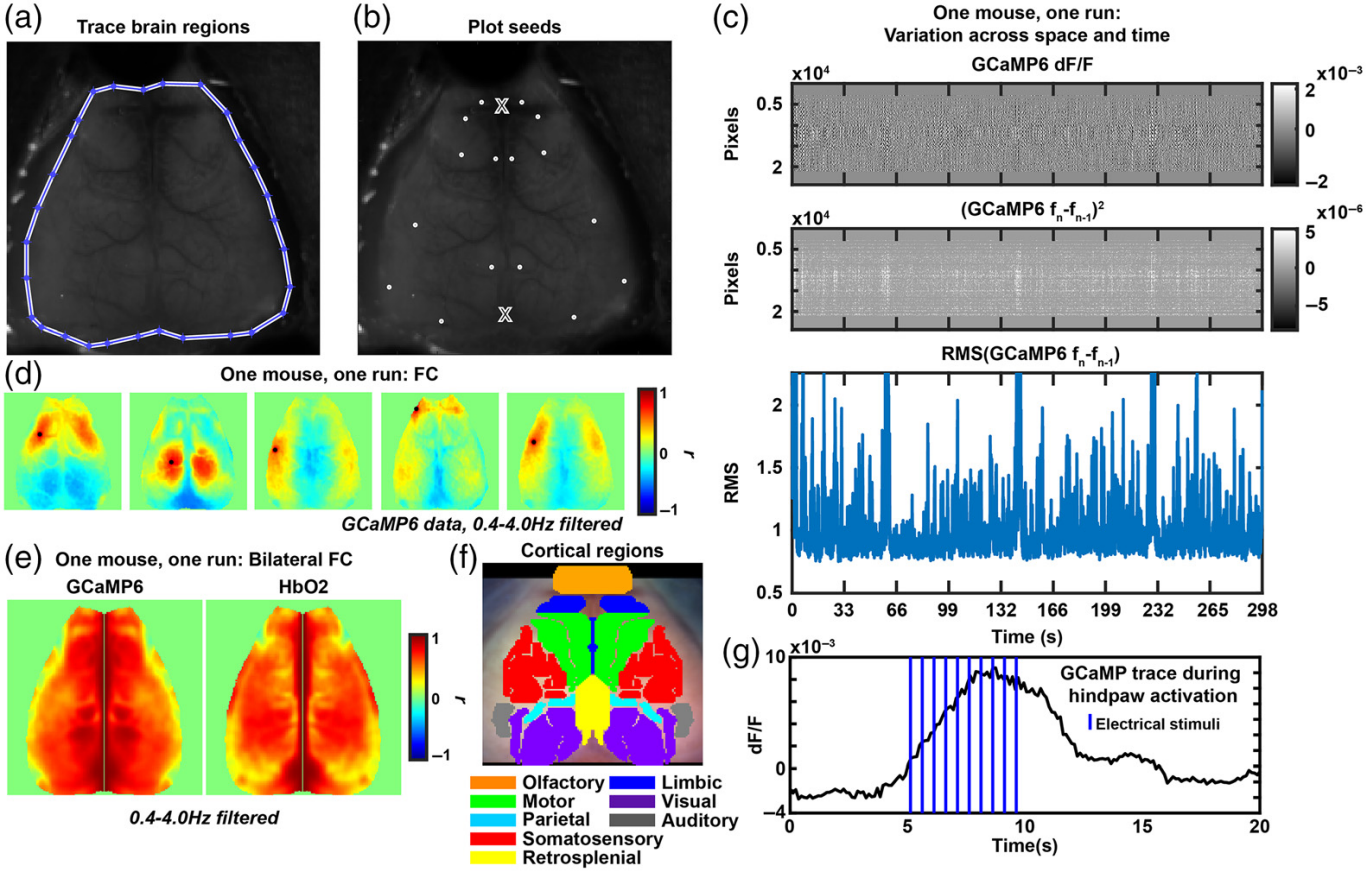
Example processing and analysis step outputs. (a) A traced dorsal cortical surface. (b) Seeds that are automatically plotted after selecting the anterior suture and lambda (marked by X). (c) Plots of pixels by time, the squared temporal derivative, or the RMS of the temporal derivative to visualize fluctuations in GCaMP6 signal during the experiment. (d) Example GCaMP6 seed-wise FC maps in one mouse, one run. (e) Example bilateral FC maps in one mouse, one run. (f) An overlaid cortical parcellation to visualize cortical functional regions. (g) A GCaMP6 activation trace using the same experimental paradigm as in Fig. 5(a) in one mouse and one experimental run. Signal was averaged across pixels in the activation area and vertical lines indicate the stimulus presence.

Within the “START” folder at https://github.com/brierl/Mouse_WOI/tree/main/START, the folder “Proc” contains the first script that should be used to load data in [following the flowchart in Fig. 1(a)]. The script load_data.m allows the user to load the image stack and normalize one frame (variable “frame5”) that will be used in the next script make_mask_landmarks.m. Within make_mask_landmarks.m, the built-in MATLAB function roipoly.m is used to prompt the user to create a binary mask representing brain regions (i.e., 1) or nonbrain regions (i.e., 0) saved to the variable “isbrain.” The user is to click along the perimeter of the brain displayed in the frame and double click upon closing the loop to create the binary file [example in Fig. 2(a)]. This file will be used later and multiplied through the image stack so only pixels corresponding to brain is analyzed within the FOV. Next, the frame is passed to the subroutine MakeSeedsMouseSpace.m within make_mask_landmarks.m where the user is prompted to click on the anterior midline suture landmark and then the lambda landmark. From these two coordinates, a prototype of seed regions used for FC analysis [explained below and example in Fig. 2(b)] is displayed on the dorsal cortical surface (corresponding to motor, somatosensory, visual cortices etc.). A box will pop up prompting the user to specify if they are content with the seed prototype. A “yes” is appropriate if the seeds appear symmetrical across midline and extend across the entire dorsal cortical surface. A “no” will prompt the user to identify the aforementioned landmarks again with the hope of creating a more symmetrical seed prototype. These landmarks are later used to affine transform images to a common image space. Herein end the steps that rely on prompted user input [outlined in Fig. 1(a)].

### 2.6 Toolbox Capabilities and Workflow: Data Processing

The data processing functionality of the toolbox represents our standard image processing pipeline, which has incorporated methodologies across multiple WOI groups and is outlined continuing in Fig. 1(b). The first processing script that should be run is proc1_sys_dep.m and outlines the processing steps that are optical system dependent. First, a frame of ambient baseline light levels collected off of the imaging system (Dark.tif, will need to be replaced on each imaging system although ours is available on GitHub) is subtracted from the time series data in the subroutine subtract_dark_ois.m. Dark.tif was created by averaging dark frames collected over 1 min. Data is then spatially and temporally detrended using the built-in MATLAB detrend.m function within the subroutine detrend_ois.m to account for global artifactual fluctuations in the data by space and time. Examples of global fluctuations affecting the time-series light levels include photobleaching, LED current drift, and nonuniformities introduced by skull anatomy. Next, the processing stream diverges based on whether data is being processed into fluctuations in hemoglobin or fluorescence data. First, for hemoglobin data, channels that will be solved for fluctuations are sent to the subroutine procPixel.m where the logmean.m function takes the pixel-wise log-ratio of each frame (raw intensity data, $F_{\text{trace}}$, over mean intensity data),

$$\log(\frac{F_{\text{trace}}}{\text{mean}(F_{\text{trace}})}),\;$$ (1)

and then the subroutine dotspect.m solves the modified Beer–Lambert law to yield fluctuations in oxygenated and deoxygenated hemoglobin, as previously explained.^7^ For fluorescence data, the respective channel is mean normalized (mean_normalize.m) and then the result is corrected for absorptions of excitation and emission light by hemoglobin fluctuations^19^ (hgb_correction.m). Following this correction, the logmean of the data is taken (logmean_fluor.m) and then the fluorescence and hemoglobin data is concatenated into a single variable, “all_contrasts.”

The next data processing script is Proc2.m and outlines the steps that are optical system independent [Fig. 1(c)]. The first subroutine, smoothimage.m, spatially smooths the data with a 5×5 Gaussian filter (1.3 pixel standard deviation). The global signal, computed by taking an average of all time traces within the FOV, gets regressed from the time series data to reduce global sources of variance such as motion and pulse, thus enhancing network correlation specificity (subroutine gsr.m).

Figure 1(d) outlines the optional processing steps. Affine.m allows for cross-mouse averaging by affine transforming the data to common Paxinos atlas space using the previously determined landmarks. The last processing script (OIS_GCaMP_Filter.m) allows for optional temporal filtering. Here, calcium data were filtered with a 0.4 to 4.0 Hz Butterworth bandpass filter and hemoglobin data with a 0.009 to 0.08 Hz Butterworth bandpass filter. These frequency bands were selected as they correspond to delta (0.4 to 4.0 Hz) and infraslow (0.009 to 0.08 Hz) ranges. The final processing script allows for an output of data quality (check_mvmt.m). This script outputs a pixels by time snapshot of contrast fluctuation [an example of this script run on GCaMP6 data in one run of one mouse in Fig. 2(c)]. The final panel is a calculation of the global variance in the temporal derivative, a surrogate measure of artifact tone as determined in human diffuse optical tomography.^20^

### 2.7 Toolbox Capabilities and Workflow: Analyses

Multiple types of analyses are supported by this toolbox and we walkthrough the two we use to analyze the stroke model in Fig. 1(e) and Table 2 (seed-based FC) and Fig. 1(f) and Table 3 (bilateral FC) post data processing. To initiate seed-based FC, folder “FC” has calc_fc.m, which takes in the processed data (e.g., “all_contrasts”), mask “isbrain,” and a seed set “seedcenter” to extract time traces within each seed region (subroutine P2strace.m) and calculate Pearson r correlation coefficients between each time trace and the remaining pixels in the FOV (subroutine strace2R.m). The next script, visualize_fc.m plots each seed-based FC map [example output in Fig. 2(d)].

**Table 2:** Seed-wise FC analysis functions to be run by user. Table of functions to calculate seed-wise FC maps as outlined in Fig. 1(e).

Function	Description
**calc_fc.m**	Calculates Pearson correlation per seed set per run
**visualize_fc.m**	Visualize seed-wise FC maps per run

**Table 3:** Bilateral FC analysis functions to be run by user. Table of functions to calculate bilateral FC maps as outlined in Fig. 1(f).

Function	Description
**calc_bilateral.m**	Calculate bilateral FC maps per run
**visualize_bilat.m**	Visualize bilateral FC maps per run

For bilateral FC calculations, start with calc_bilateral.m within folder “BilatFC,” which requires the same data and mask inputs as calc_fc.m (note: this calculation does not need a seed set). The subroutine CalcRasterSeedsUsed.m outputs a matrix of left hand and right hand side pixel pairs that a Pearson r correlation value will be calculated between. Subroutines fcManySeed.m and CalcBilateral.m calculates the Pearson r value and organizes the r matrix into an image across the dorsal cortical surface. The next script, visualize_bilat.m, outputs a figure as shown in Fig. 2(e) of the bilateral FC maps for each contrast analyzed. A parcellated dorsal cortical surface is provided in Fig. 2(f) to orient users to the cortical regions within the FOV.

Additional analysis supported by the toolbox includes stim activation plotting and tracing [outlined in Fig. 1(g) and Table 4] in folder “Stims.” The user should run calc_stims.m with data (“all_contrasts”) and mask (“isbrain”) inputs. This script has commented variables to define the length of stimulus blocks so that the data can be rearranged into pixels by pixels by stimulus block length by number of blocks. Then, a mean frame (calculated by averaging together frames when the stimulus is off) is subtracted from the time-series data. The script plots an average frame from when the stimulus is on and thresholds the image at 80% max activation. The pixels surviving this threshold are averaged spatially, and the trace across averaged stimulus blocks is plotted [example trace for one mouse in Fig. 2(g)].

**Table 4:** Stimulus activation analysis function to be run by user. Function to calculate stimulus activation maps and traces as outlined in Fig. 1(g).

Function	Description
**calc_stims.m**	Calculate average activation maps and activation traces. Visualize both

Outlined in Fig. 1(h) and Table 5 are the scripts used to perform the cluster size-based statistical thresholding (in folder “Stats”) described below. The user should run cluster_threshold.m, which requires data (“all_contrasts”), a mask (“isbrain”), and an alpha level [the overall family-wise error (FWE) rate e.g., p=0.05] as inputs. The script calls subroutine FWHM_ParDer.m, which estimates the FWHM based on a covariance matrix of the spatial partial derivatives of the data. Then, the threshold for cluster size significance is solved for and output.

**Table 5:** Cluster statistical analysis function to be run by user. Function to calculate cluster size for statistical significance as outlined in Fig. 1(h).

Function	Description
**cluster_threshold.m**	Solve for cluster size-based threshold

To enhance visualization, all of these maps can be plotted onto our cortical parcellation map (as in Fig. S2 in the Supplementary Material) as determined by White et al.,^7^ using the Parcellations.mat file (variable “Parcels”) located in the “START” folder.

### 2.8 Electrical Hindpaw Stimulus Imaging

A stimulus driven protocol was used to further show the utility of the statistical correction method described. Two GCaMP6 mice were surgical prepared as previously described. Mice were anesthetized and microvascular clips were applied to the left hindpaw. An isolated pulse stimulator was used to deliver 2 Hz, 300  μs, 0.5 mA electrical stimuli. Each imaging block consisted of 5 s rest, 5 s stimuli, and 10 s rest. Each mouse underwent 14 imaging blocks. GCaMP6 data were processed as described above and spatially downsampled to 78 pixels^2^ and temporally downsampled to a framerate of 8.4 Hz. No temporal filtering was used and the pixel-wise average time trace was subtracted. Average activation maps were calculated by averaging across imaging blocks then across mice. A one-sample t-test was used to compare activation areas to the null hypothesis of no activation areas.

### 2.9 Cluster-Based Thresholding

**Fig. 3:**
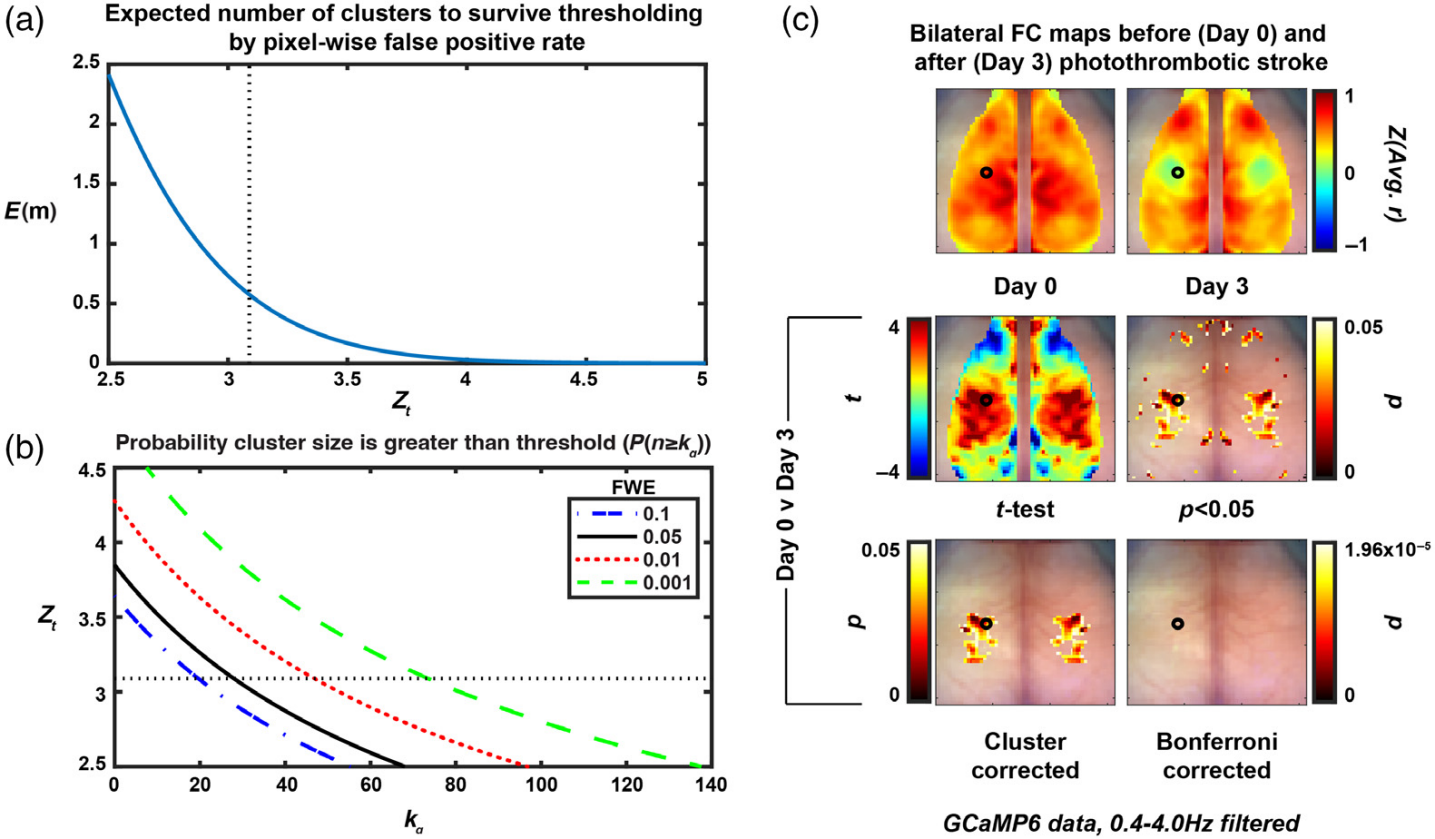
Random field theory identifies cluster size threshold for pixel-wise t-maps on GCaMP6 bilateral FC data. (a) The relationship between the pixel-wise false positive rate (determined by $Z_{t}$, vertical dashed black line is $Z_{t}=3.09$, which corresponds to p=0.001) and the expected number of clusters to survive thresholding due to chance (E[m]). (b) The relationship between the pixel-wise false positive rate (determined by $Z_{t}$, horizontal dashed black line is $Z_{t\;}=3.09$, which corresponds to p=0.001), the family-wise error rate (here, 0.05) and cluster size needed for significance. (c) Top row: average (N=4) bilateral FC maps pre (left) and 3 days post (right) photothrombotic stroke to left somatosensory forepaw cortex (marked by hollow black circle). Middle row: pixel-wise paired t-test (left) and thresholded image for pixels with p<0.05 (right). Bottom row: thresholded image with FWE = 0.05 using the cluster size-based method (left) and image with Bonferroni correction for multiple comparisons (right).

A challenge with analyzing the statistical significance in functional imaging is managing the multiple comparisons problem. Here, we used a cluster size-based method that leverages the spatial connection between pixels and credits large clusters as having more statistical significance than small clusters with the same peak t-value. More specifically, we used a cluster size-based thresholding method (determined by the cluster-size limit, $k_{\alpha }$) to analyze two-dimensional FC maps and to ensure the FWE rate did not exceed 5%. A random field theory (RFT) approach was adapted from the fMRI and DOT literature^15^^,^^16^ to fit our two-dimensional data. Using RFT, we are able to approximate the expected (E[ ]) number of clusters (m) in an image at a given z-score threshold [$Z_{t}$, Fig. 3(a)]:

$$E[m]=R*4\;\ln(2)*2\pi^{-3/2}*Z_{t}e^{-\frac{1}{2}*Z_{t}^{2}},$$ (2)

which holds true for increasing values of $Z_{t}.$ Here, we use $Z_{t}=3.09$, which corresponds to a false positive rate of 0.001 at the pixel level. R represents the number of resolution elements provided by the optical system:

$$R=x^{2}/{\mathrm{FWHM}}^{2},$$ (3)

where x is the number of pixels in the image in one dimension and FWHM is the full-width half maximum of the point spread function estimated from the covariance of the spatial partial derivatives of a fully processed image.^16^^,^^21^ Here, we use x=78  pixels (156 pixel^2^ images that have been downsampled by 2, 11 mm total) and calculate an FWHM of about 7 pixels (0.99 mm) for the imaging system data. The FWHM is solved for using one frame of an image stack within the subroutine FWHM_ParDer.m as detailed by Hassanpour et al. and Xiong et al.^16^^,^^21^ Briefly, a covariance matrix is calculated on the spatial partial derivatives of one frame and the relationship between this covariance matrix (Λ) and the FWHM is defined as

$$|\Lambda |={\left[\frac{{\mathrm{FWHM}}^{2}}{4\;\ln(2)}\right]}^{-2}.$$ (4)

We are able to then approximate the expected number of pixels (N) above $Z_{t}$ as

E[N]=E[m]*E[n], (5)

where n is the pixel count within a cluster and

$$E[n]=\frac{2\pi {\mathrm{FWHM}}^{2}}{Z_{t}^{2}4\;\ln(2)}.$$ (6)

The probability that n will exceed any threshold, x, can be modeled by the exponential function

$$P(n\geq x)=e^{-\beta x},$$ (7)

where β can be expressed as

$$\beta =\frac{\Gamma (2)E[m]}{E[N]},$$ (8)

where Γ is the gamma function. Substituting Eq. (6) into Eq. (5) and then the newly formed Eq. (5) into Eq. (8) rearranges the above to

$$\beta =\frac{\Gamma (2)Z_{t}^{2}4\;\ln(2)}{2\pi {\mathrm{FWHM}}^{2}}.$$ (9)

Providing an equation (with all previously solved for values) that varies with the inverse of the squared FWHM. We only want clusters to survive thresholding with a family-wise error rate of 0.05 (α), therefore, we want to find the pixel count threshold, $k_{\alpha }$, that would result in at least one cluster surviving threshold when there is no true significant difference, 5% of the time. Essentially, we are asking for a $k_{\alpha }$ where the following is true:

$$P(n\geq k_{\alpha })=\alpha .$$ (10)

Assuming no clusters are a true positive. Which, mathematically speaking, is the same as solving for 1 minus the probability that no clusters have a pixel count above threshold $k_{\alpha }$ [Fig. 3(b)]:

$$P(n\geq k_{\alpha })=\overset{\infty }{\underset{i=1}{\sum }}P(m=i)[1-P{\left(n<k_{\alpha }\right)}^{i}],$$ (11)

$$=1-e^{-E[m]*P(n\geq k_{\alpha })},$$ (12)

which yields:

$$k_{\alpha }=\frac{1}{\beta }\;\ln(\frac{-E[m]}{\ln(1-\alpha )}).$$ (13)

After substituting Eq. (7) into Eq. (12) and solving for $k_{\alpha }$.

Pixel-wise t-tests were performed to compare Day 0 and Day 3 stroke FC maps, and to compare stimulus activated maps to the null hypothesis of no activation. These t-test maps were then thresholded, leaving pixels with a t-value corresponding to p<0.05. If the number of pixels in the remaining clusters exceeded $k_{\alpha }$, these clusters were considered to have a statistically significant finding within them by the cluster size-based technique.

### 2.10 Data and Code Availability

To promote validation and comparative analyses by external groups, data and specific code will be made available through requests to the corresponding author. The toolbox presented here, (for WOI processing and analysis) is available online (https://github.com/brierl/Mouse_WOI/tree/main/START/) and was used for all present analysis. Stroke and stim data is available on Mendeley Data (stroke^22^ and stim^23^) and detailed in Table 6.

**Table 6:** Sample data available on Mendeley Data.^22^^,^^23^ Rows 1–4: table of the sample unprocessed data available online corresponding to two, 5-min baseline and two, 5-min post stroke runs. Rows 5 and 6: sample processed 5-min stim data runs in 14 blocks of 20 s each broken down into 5 s of rest, 5 s of 2 Hz stims, and 10 s of rest.

Function	Description
Pixels × pixels × frames, resting state, 5 min, Hr0	201118-1253-1-fc1.mat
Pixels × pixels × frames, resting state, 5 min, Hr0	201118-1254-5-fc1.mat
Pixels × pixels × frames, resting state, 5 min, Hr72	201122-1253-1-fc1.mat
Pixels × pixels × frames, resting state, 5 min, Hr72	201122-1254-5-fc1.mat
Pixels × pixels × frames × block, stim, 5 min	112233-TestMs3-stim1-Affine_GSR_BroadField_Stim.mat
Pixels × pixels × frames × block, stim, 5 min	112233-TestMs4-stim1-Affine_GSR_BroadField_Stim.mat

## 3 Results

### 3.1 Toolbox Overview

We seek to distribute a comprehensive, easy-to-use, open-source toolbox for mouse WOI data processing and analysis. Within this toolbox, we have translated multiple techniques from the human fMRI literature to the WOI mouse world. The toolbox is largely split up into two pipelines (Fig. 1, Tables 1–5), data processing and postprocessing analysis. A walkthrough is provided in Sec. 2, but briefly, prompted user input is needed while running scripts to load data and mark brain regions and anatomical landmarks [Figs. 1(a), 2(a), and 2(b)]. The remaining processing steps outlined in Figs. 1(b)–1(d) are split up into optical system dependent (for spectroscopy and fluorescence data processing) and independent steps, as well as optional steps [Fig. 2(c)]. After processing the data, multiple analysis pipelines are supported. Figures 1(e)–1(h) outlines the steps for the analyses shown in later figures, namely FC calculations [Figs. 2(d) and 2(e)], stimulus activation plotting [Figs. 2(f) and 2(g)], and statistical testing.

### 3.2 Cluster Size-Based Thresholding Applied to Stroke Data

The cluster size-based statistical thresholding method^15^^,^^16^ is detailed in the Methods and was adapted from the fMRI and DOT literature. The method is used here to select clusters of size $k_{\alpha }$ (expressed in number of pixels) with p<0.05 by the pixel-wise paired t-test method that satisfies the pixel-wise false positive rate (set by $Z_{t}$) and overall family-wise error rate [Figs. 3(a) and 3(b)]. Using this cluster size cutoff, we were able to compare bilateral FC maps at baseline (N=4, Day 0) and 72 h post (N=4, Day 3) photothrombotic stroke [Fig. 3(c)]. Photothrombosis was induced in left somatosensory cortex, which resulted in loss of homotopic FC at Day 3. A pixel-wise t-test was performed and thresholded to only display regions with p<0.05 (note, this map is not corrected for multiple comparisons). Using the cluster-size based threshold (FWE = 0.05), we were able to localize a somatosensory anchored deficit. This deficit was overlaid and contained completely within the parcellated somatosensory cortex (Fig. S2 in the Supplementary Material). Due to the symmetrical nature of the bilateral FC calculation, loss of homotopic FC is visualized by a symmetrical deficit [green area in Fig. 3(c) upper right panel]. Using the Bonferroni correction for multiple comparisons, no regions survived this stringent cutoff, resulting in no significant differences between day 0 and day 3 with this method.

**Fig. 4:**
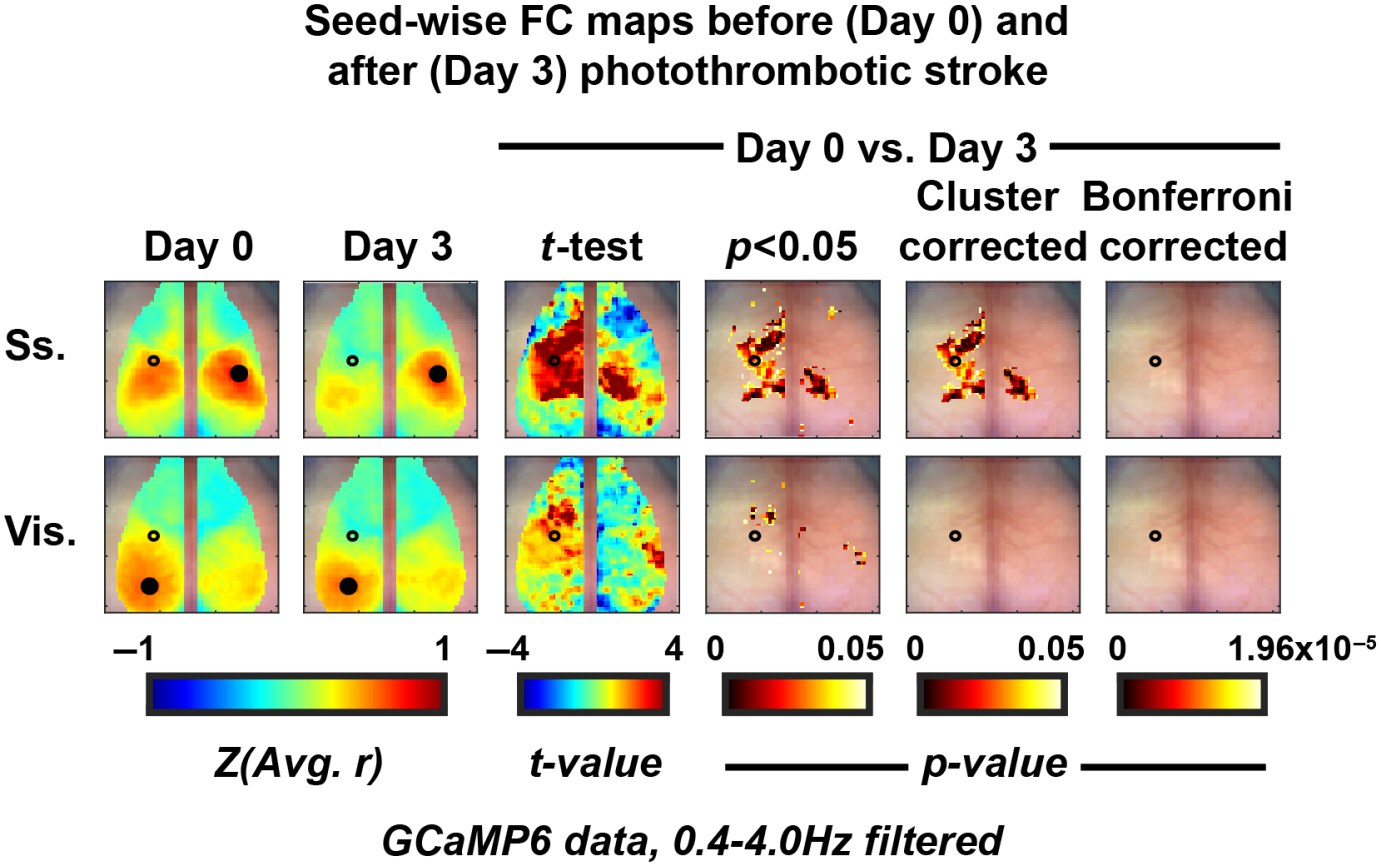
Cluster size thresholding yields Ss based deficit and unaffected visual cortex using a seed-wise FC methodology with calcium data. Average (N=4) seed-wise FC maps using seeds in somatosensory (Ss.) and visual (Vis.) cortices at (first column) baseline and (second column) 3 days post photothrombotic stroke to left somatosensory forepaw cortex (marked by hollow black circle). Third column: pixel-wise paired t-test between Day 0 and Day 3. Fourth column: thresholded images for pixels with p<0.05. Fifth column: thresholded images showing regions of statistically significant change using a clustered-based methodology with FWE = 0.05. Sixth column: no pixels survived the Bonferroni correction for multiple comparisons.

Further exploring cross-network alterations in the context of this stroke model, we calculated seed-wise FC maps at Day 0 and Day 3 (Fig. 4). Using the same statistical framework, we compared the results at Day 0 and Day 3 and illustrated significant network alterations bilaterally in the seed-wise somatosensory (Ss.) map and an unaffected visual cortex outside of the area of photothrombosis.

Repeating the same analysis and using oxygenated hemoglobin as a contrast, we see a similar deficit within somatosensory cortex in the seed-wise FC maps (Fig. S1 in the Supplementary Material). However, with hemoglobin as a contrast, we lose statistical significance in this experiment as the somatosensory-based deficit does not survive cluster-based statistical thresholding. Additionally, regions of cortex survive our cluster-based statistical analysis when using a visual seed contrary to when this analysis was performed using calcium.

### 3.3 Cluster Size-based Thresholding Applied to Stimulus Data

**Fig. 5:**
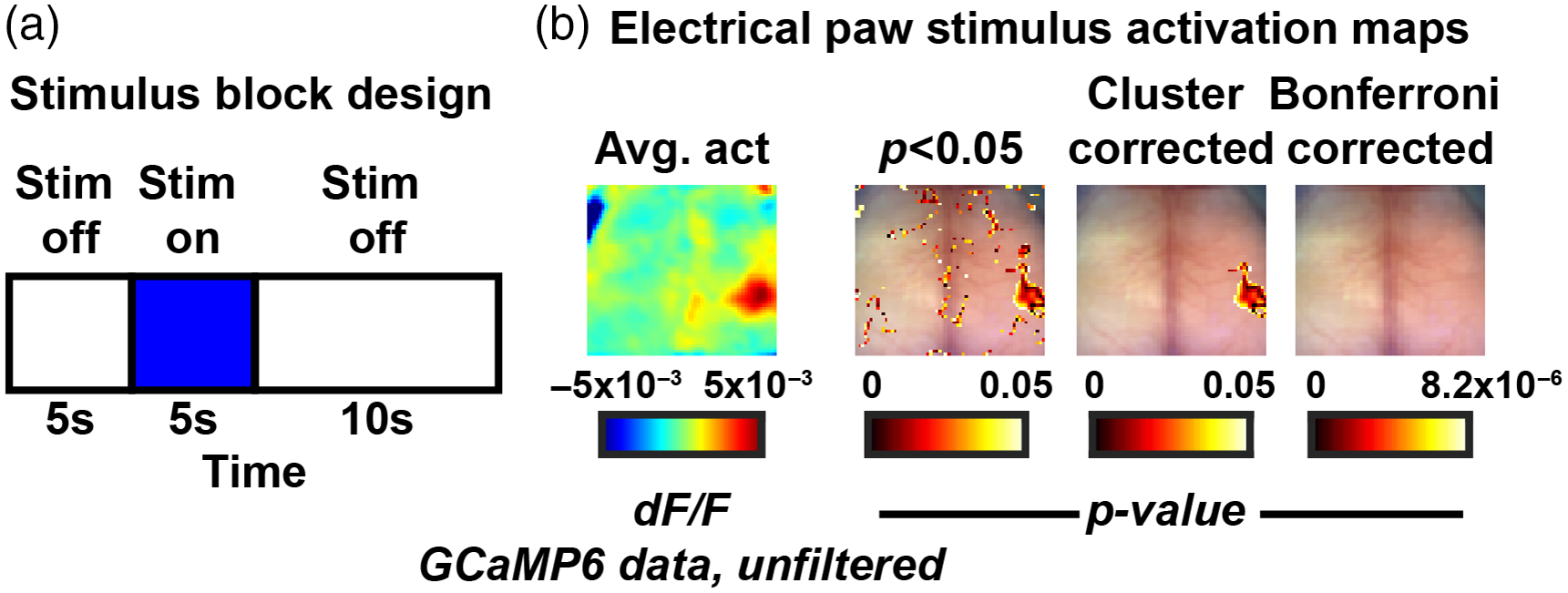
Cluster size thresholding plots the GCaMP6 cortical activation in response to electrical sensory stimulus. (a) The experimental paradigm consisting of 5 s of rest, 5 s of stimulus on, 10 s of rest, that is repeated throughout the imaging duration. (b) Temporally averaged frame across durations of stimulus on (left) followed by a one-sample t-test thresholded for areas of p<0.05 and respective cluster-based and Bonferroni corrections.

We imaged two GCaMP6 mice that underwent anesthetized electrical hindpaw stimuli experiments. The protocol consisted of multiple blocks of 5 s of rest, 5 s of 2 Hz stimulus, and 10 s of rest [Fig. 5(a)]. The average activation while the stimulus was delivered was mapped [Fig. 5(b)]. A one-sample t-test was thresholded for p<0.05 leaving multiple areas of cortex. A Bonferroni correction for multiple comparisons removed all activation areas while the cluster-based correction method localized the somatosensory-based activation while removing spurious artifacts.

## 4 Discussion

WOI, especially of mice expressing genetically encoded calcium indicators (GECIs), provides cell-specific, improved temporal resolution recordings of calcium transients across the entire mouse cortex.^8^^–^^10^ Functional neuroimaging analysis pipelines have been thoroughly developed in the functional magnetic resonance imaging (fMRI) literature,^24^^,^^25^ however, despite having many similarities, these analytical techniques have not been translated into a comprehensive, user friendly mouse WOI toolbox. Here, we provide a toolbox that processes data according to previous reports.^8^^,^^19^ We also implement some statistical approaches from the fMRI literature to handle the massive multiple comparisons problem present in all functional neuroimaging data.

The spatial resolution afforded by techniques such as fMRI or WOI allow neuroscience inquiries with network-level spatial specificity.^2^^,^^9^ However, treating each voxel or pixel as an independent measure will result in an almost always insurmountable correction for multiple comparisons (e.g., using the Bonferroni correction). Therefore, there has been significant work done in the fMRI literature to develop more appropriate algorithms to address this.^15^ The cluster extent-based thresholding statistical approach operates on the hypothesis that neighboring pixels are likely not independent samples (i.e., a Bonferroni correction for multiple comparisons would be too stringent). Therefore, large-grouped differences via independent pixel-wise statistical tests are more likely to represent a significant change somewhere within that cluster. Here, this method is set up to have a family-wise error (FWE) rate of 5%, meaning in the collection of thresholded pixel-wise t-tests, there is a 5% chance of having at least one false positive result. An advantage of this technique is that it allows for spatial specificity of activation or change in connectivity by considering all pixels or voxels in the FOV. However, this method only works as well as the initial analysis does in specifically isolating an activation or change since the rightful conclusion of a cluster surviving thresholding is that a change or activation occurred within that cluster. Here, we translate this method used in three-dimensional fMRI to two-dimensional WOI data and demonstrate the ability to localize a somatosensory-based deficit 3 days after a photothrombotic event to left somatosensory cortex [Fig. 3(c)]. Using a seed-wise approach to calculating FC, we were able to illustrate this same somatosensory anchored deficit (note the bilateral deficit when using the Ss. Seed, Fig. 4). Notably, both hemispheres are affected using this seed-wise approach, with a larger surface area of deficit on the left (where the area of photothrombosis occurred) as opposed to the right. Further, we illustrate the spatial specificity of the analysis when using calcium dynamics through an unaffected visual cortex outside of the area of photothrombosis. This was in contrast to the hemoglobin-based dynamics run through the same analysis where we did unexpectedly find deficits using a visual seed due to a somatosensory stroke (Fig. S1 in the Supplementary Material, visual seed). It is evident when examining the Day 0 and Day 3 FC maps that there is an extra level of spatial specificity afforded by the calcium mapping, supporting our claim that the cluster-based statistical thresholding methodology is dependent on the spatial specificity of the initial analysis. Additionally, there was more variability in the hemoglobin-based measurements, as displayed by the smaller t-values and subsequent loss of statistical significance despite the large effect size in the somatosensory FC map.

Optical imaging is often burdened by spurious artifact at the edges of the FOV. There is a precedence for discarding these regions; however, this statistical correction method supports this in a data-driven and mathematical manner. Our stimulus experiment purposefully used a small sample size (N=2) to show off the utility of the statistical package. It was apparent though, that due to the small sample size, there were multiple areas surviving initial t-testing that were not due to the experimental protocol (Fig. 5). Some of these areas were at the edge of the FOV but some were not. Our methodology here provides an unbiased way to handle the imaging data to correctly map regions of activation (stimulus) or deficits (stroke).

## 5 Conclusions

This toolbox fills a much-needed gap between the fMRI and WOI data processing communities. Shown throughout are examples of a commonly used statistical measure that was developed within fMRI being applied to FC and stimulus activations calculated with the WOI data. However, the toolbox is also set up to compute node degree,^26^ spectral content,^9^ multivariate FC,^27^ and a neurovascular coupling (NVC) approximation,^8^ which can all be plotted topographically and corrected via cluster-based thresholding (these packages also available at https://github.com/brierl/Mouse_WOI). The wide distribution and use of this toolbox will greatly aid groups that are hoping to start imaging mouse models of health and disease to better understand how brain dynamics might change in humans.
