# Supplementary material for: Open-source statistical and data processing tools for wide-field optical imaging data in mice
Source: Neurophotonics. 2023 Mar 1;10(1):016601. doi: 10.1117/1.NPh.10.1.016601 (PMC9976616; doi:10.1117/1.NPh.10.1.016601)
Supplement: Supplementary file 1 [file NPh_010_016601_SD001.pdf]

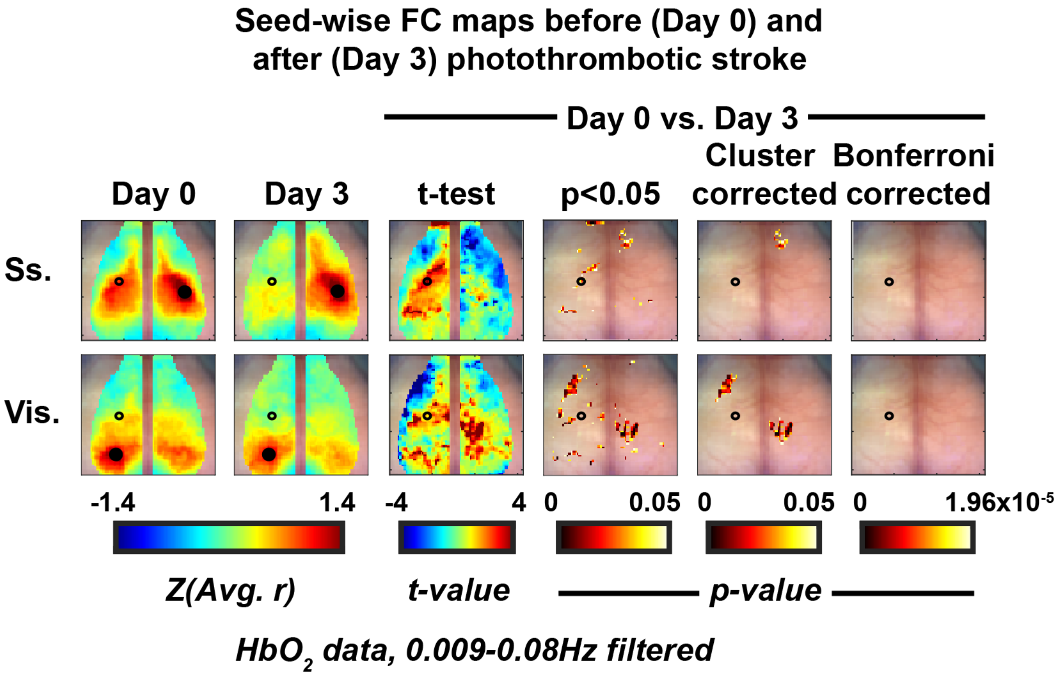

Figure S1: Cluster size thresholding does not detect a Ss based deficit with hemoglobin data. Average (N=4) seed-wise FC maps using seeds in somatosensory (Ss.) and visual (Vis.) cortices at (1st column) baseline and (2nd column) 3 days post photothrombotic stroke to left somatosensory forepaw cortex (marked by hollow black circle). (3rd column) Pixel-wise paired t-test between Day 0 and Day 3 and thresholded image for pixels with $p<0.05$ (4th column). (5th column) Thresholded images showing regions of statistically significant change using a clustered-based methodology with FWE=0.05. (6th column) No pixels survived the Bonferroni correction for multiple comparisons.

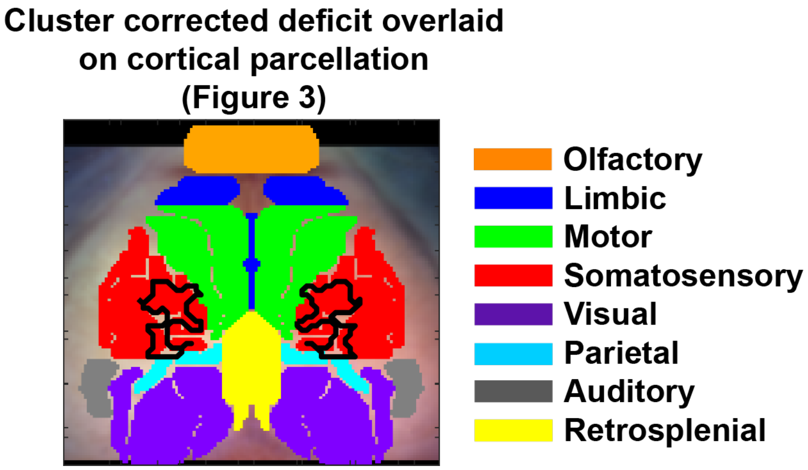

Figure S2: Ss based deficit from Figure 3 overlaid onto a cortical parcellation.
